# A novel lightweight YOLOv8-PSS model for obstacle detection on the path of unmanned agricultural vehicles

**DOI:** 10.3389/fpls.2024.1509746

**Published:** 2024-12-24

**Authors:** Zhijian Chen, Yijun Fang, Jianjun Yin, Shiyu Lv, Farhan Sheikh Muhammad, Lu Liu

**Affiliations:** ^1^ School of Agricultural Engineering, Jiangsu University, Zhenjiang, China; ^2^ Institute of Technology, Anhui Agricultural University, Hefei, China

**Keywords:** UAV, YOLOv8, depth camera, obstacle detection, PConv, slim-neck, shape-IoU

## Abstract

**Introduction:**

The rapid urbanization of rural regions, along with an aging population, has resulted in a substantial manpower scarcity for agricultural output, necessitating the urgent development of highly intelligent and accurate agricultural equipment technologies.

**Methods:**

This research introduces YOLOv8-PSS, an enhanced lightweight obstacle detection model, to increase the effectiveness and safety of unmanned agricultural robots in intricate field situations. This YOLOv8-based model incorporates a depth camera to precisely identify and locate impediments in the way of autonomous agricultural equipment. Firstly, this work integrates partial convolution (PConv) into the C2f module of the backbone network to improve inference performance and minimize computing load. PConv significantly reduces processing load during convolution operations, enhancing the model's real-time detection performance. Second, a Slim-neck lightweight neck network is introduced, replacing the original neck network's conventional convolution with GSConv, to further improve detection efficiency and accuracy. This adjustment preserves accuracy while reducing the complexity of the model. After optimization, the bounding box loss function is finally upgraded to Shape-IoU (Shape Intersection over Union), which improves both model accuracy and generalization.

**Results:**

The experimental results demonstrate that the improved YOLOv8_PSS model achieves a precision of 85.3%, a recall of 88.4%, and an average accuracy of 90.6%. Compared to the original base network, it reduces the number of parameters by 55.8%, decreases the model size by 59.5%, and lowers computational cost by 51.2%. When compared with other algorithms, such as Faster RCNN, SSD, YOLOv3-tiny, and YOLOv5, the improved model strikes an optimal balance between parameter count, computational efficiency, detection speed, and accuracy, yielding superior results. In positioning accuracy tests, the, average and maximum errors in the measured distances between the camera and typical obstacles (within a range of 2-15 meters) were 2.73% and 4.44%, respectively.

**Discussion:**

The model performed effectively under real-world conditions, providing robust technical support for future research on autonomous obstacle avoidance in unmanned agricultural machinery.

## 1 Introduction

The fast rate of urbanization, along with an aging rural population and migration of agricultural workers, has resulted in a considerable labor shortage in rural regions. These issues make it increasingly difficult to sustain high-quality farming techniques while failing to fulfill the expectations of modern agricultural growth. As a result, the automation of agricultural technology has become an absolute requirement for agricultural advancement. At present, such difficulties can be effectively solved through the development of smart agriculture (Garg and Alam, 2023) and unmanned farms are an important way to realize smart agriculture (Ming et al., 2023). Many developed countries in Europe and the United States have streamlined operations using unmanned farm technologies such as automated navigation for smart farm machinery (Yang et al., 2022; Ji et al., 2023). Compared to conventional agricultural production techniques, automation can increase operating hours, reduce operator labor intensity, and enhance overall efficiency (Wang et al., 2022). However, the unstructured features of agricultural landscapes create considerable obstacles. The random and discrete distribution of obstacles, such as pedestrians in the field or other agricultural machinery operating simultaneously, poses serious risks to the autonomous navigation of agricultural equipment. Accurate obstacle detection during operation is essential for effective path planning and obstacle avoidance (Zhao et al., 2023). It is significant to ensure the operational efficiency and driving safety of agricultural machinery.

Obstacle detection can be achieved by employing various obstacle detection sensors, including vision sensors (Fu et al., 2020)、laser radar (Yang et al., 2022)、and ultrasonic sensors (Li et al., 2018). Among these methods, vision-based detection methods have the advantages of low price, rich information, and wide detection range, which are widely used in theoretical research and practical application exploration. Traditional target detection algorithms rely on feature extraction and sliding window techniques to identify specific objects in an image and determine their location (Dong et al., 2023). However, in complex and dynamic environments manually extracted features may not effectively describe and distinguish, objects, affecting the accuracy and robustness of detection. With the rise of deep learning technology, target detection algorithms based on this technology have gradually replaced traditional algorithms, becoming mainstream, emerging and widely used in the agricultural field in recent years (Ren et al., 2016; Ji et al., 2022). These algorithms are mainly categorized into two-stage target detection algorithms represented by Faster R-CNN and one-stage target detection algorithms SSD and YOLO detection algorithms, which are now widely used in target detection (Wang et al., 2021). To address obstacle detection in complex orchard environments, Liu et al (Hui et al., 2019). proposed a real-time pedestrian identification approach based on an enhanced SSD architecture that utilizes MobileNetV2 as the backbone. To anticipate position, the auxiliary network layer uses an inverted residual structure and null convolution. When tested on an open dataset, the upgraded SSD model obtained an average accuracy of 97.46% and a recall of 89.72% in pedestrian recognition, despite only identifying pedestrians as a single obstacle type. Chen et al (Bin et al., 2021). created an enhanced YOLOv3-tiny target detection model to facilitate in obstacle avoidance during autonomous agricultural machinery navigation. This model incorporates shallow characteristics via a splicing layer before the second prediction layer, as well as a residual module, to improve the depth and learning capacity of the YOLOv3-tiny backbone. This advancement makes it possible to use a panoramic camera installed on the agricultural machine to detect impediments in real time with greater accuracy. The results show that the average accuracy and recall rates increased by 5.6% and 5.2%, respectively, satisfying the requirement for real-time obstacle detection while moving. Wei et al (Jiansheng et al., 2021). developed an obstacle sensing system for autonomous tractors based on an upgraded YOLOv3 model for field detection and binocular vision for obstacle localization. The experimental findings demonstrated an average accuracy of 89.54%, a recall of 90.18%, a depth estimate error of 4.66% for dynamic situations, and an average processing time of 0.573 seconds. However, the large number of YOLOv3 model parameters reduces its speed on embedded systems, emphasizing the need for a more lightweight model to increase inference speed. Su et al (Su et al., 2022). enhanced the YOLOv5s model for real-time detection of orchard obstacles, such as people, by using K-means clustering, the Senet module, and pruning. Experimental findings indicated that the revised model decreased size by 13.6 MB while boosting accuracy and average accuracy by 5.60% and 1.30%, respectively. The average detection time reaches 33 ms, which meets the requirements for obstacle detection in orchards.

The above research has promoted the development of obstacle detection technology, and a certain degree of progress has been made in terms of detection accuracy, detection speed, and lightweight. However, the unstructured field environment also brings huge challenges. The obstacles in the field are not single types of objects, usually composed of static and dynamic types of obstacles. This often leads to the model ignoring some objects during detection, which poses certain safety hazards; In addition, most existing obstacle detection models are complex large models that cannot run when deployed on low performance embedded devices on agricultural machinery. Considering the safety and work efficiency of unmanned agricultural machines in the process of travelling, it is particularly important to achieve fast and accurate detection and localization of different types of obstacles. To address the problem that the complexity of the model leads to difficulties in mobile deployment, from the perspective of balancing the detection speed and detection accuracy, this paper proposes an improved network model based on YOLOv8, which achieves obstacle detection and localization after the model is deployed in the edge computer, to better leave a safe distance for the unmanned agricultural vehicle to avoid obstacles. The main contributions of our paper are summarized as follows:

- This article constructs an obstacle dataset suitable for unmanned agricultural vehicle driving scenarios by collecting images of different types of obstacles.
- In order to reduce the model size and maintain high detection speed while ensuring accuracy, this paper proposes a lightweight YOLOv8-PSS model.
- This article deploys the model on agricultural machinery and detects and locates different types of obstacles, verifying that the proposed YOLOv8-PSS model can maintain good detection performance after deployment, laying the foundation of the subsequent unmanned agricultural vehicle intelligentsia.

## 2 Materials and methods

### 2.1 Dataset creation

#### 2.1.1 Data source

**Figure 1:**
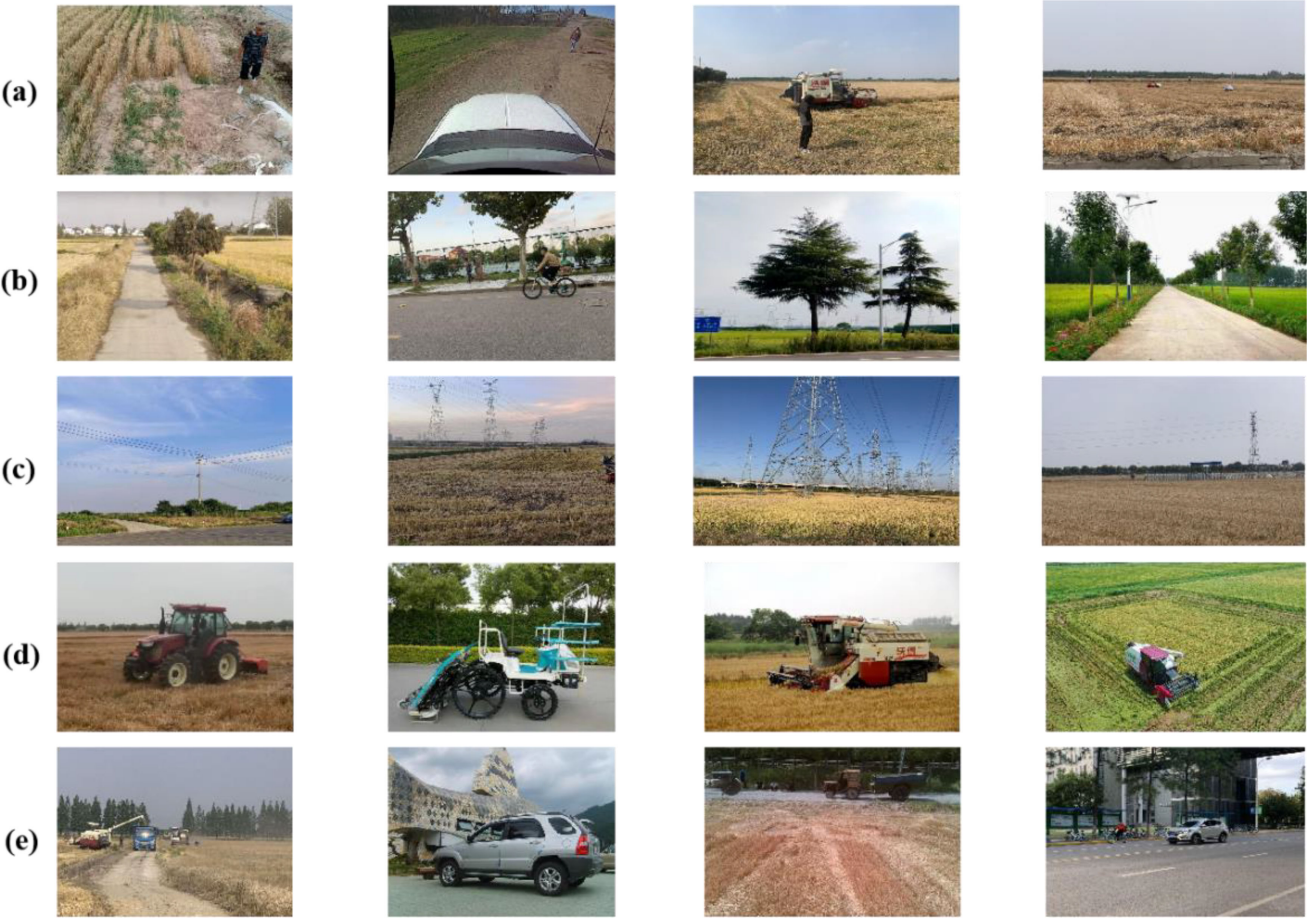
Sample of obstacles (**(A)** person, **(B)** tree, **(C)** pole, **(D)** agm, **(E)** vehicle).

To increase dataset variety and assure robust detection and generalization during real-world operations, this research combines experimental data from both open-source and self-constructed datasets. The open-source data is sourced from Carnegie Mellon University’s National Robotics Engineering Center (https://www.nrec.ri.cmu.edu/solutions/agriculture/human-detection-and-tracking-2/), Flying Plasma AI Studio’s public dataset, and images collected from the web. The self-constructed field obstacle dataset was collected from Rungo Farm Co. in Zhenjiang City, Jingkou District, Jiangsu Province, China (Longitude 119°43’55.664″ East, Latitude 32°8’12.998″ North) and Green Spring Leisure Agricultural Demonstration Park in Huimin County, Binzhou City, Shandong Province, China (Longitude 117°32’5.136″ East, Latitude 37°27’32.342″ North), between June 2023 and early November 2023. Images were captured from 8:00 to 18:00, and the acquisition equipment was an iPhone XS and Canon camcorder with lens resolutions of 3624 × 2448 and 3456 × 2304 pixels, respectively (https://www.kaggle.com/datasets/chenzhijian37/agricultural-obstacles). All data images were collected in the natural environments along farm roads, and unclear images caused by weather or human error were removed. An example of the dataset is shown in **Figure 1**, which mainly includes common obstacles such as people, farm machinery, trees, vehicles, and utility poles.

#### 2.1.2 Image pre-processing

To further enrich the dataset, this research uses online data enhancement techniques to augment the data volume, specifically including luminance degradation adjustment, random colors, random noise, etc. Considering that the obstacles are normal during the agricultural machine operation, this research does not add other data enhancement methods, such as rotation and flip. LabelImg software is used to label the obstacles in the image with the minimum outer rectangular box, and the format of the LabelImg is a Txt file in YOLO format (Ji et al., 2021; Hu et al., 2023). The dataset was randomly divided into a training set (7344 images), validation set (909 images), and test set (908 images) in a 7:2:1 ratio, as detailed in **Table 1**.

**Table 1:** Classification of different obstacle samples.

Type	Train	Validation	Test
Person	1679	190	187
Tree	1154	168	173
Pole	1226	177	175
Agm	1536	186	181
Vehicle	1749	188	192

### 2.2 YOLOv8

The YOLOv8 network, launched by Ultralytics in January 2023, introduces several improvements over the widely used YOLOv5. It consists of four main components: input, backbone, neck, and head (Yang et al., 2023). YOLOv8 officially provides a series of models in various sizes (Zhang et al., 2024), including YOLOv8n, YOLOv8s, YOLOv8m, YOLOv8l, and YOLOv8x, which differ in network width (number of channels) and depth (number of layers), aiming to satisfy the speed and accuracy requirements in different scenarios. In this research, the smallest YOLOv8n is selected as the baseline model for target detection due to its speed, stability, and efficiency. It also offers scalability, making it well-suited for real-time obstacle detection in various target detection scenarios.

### 2.3 Obstacle recognition based on lightweight YOLOv8-PSS model

**Figure 2:**
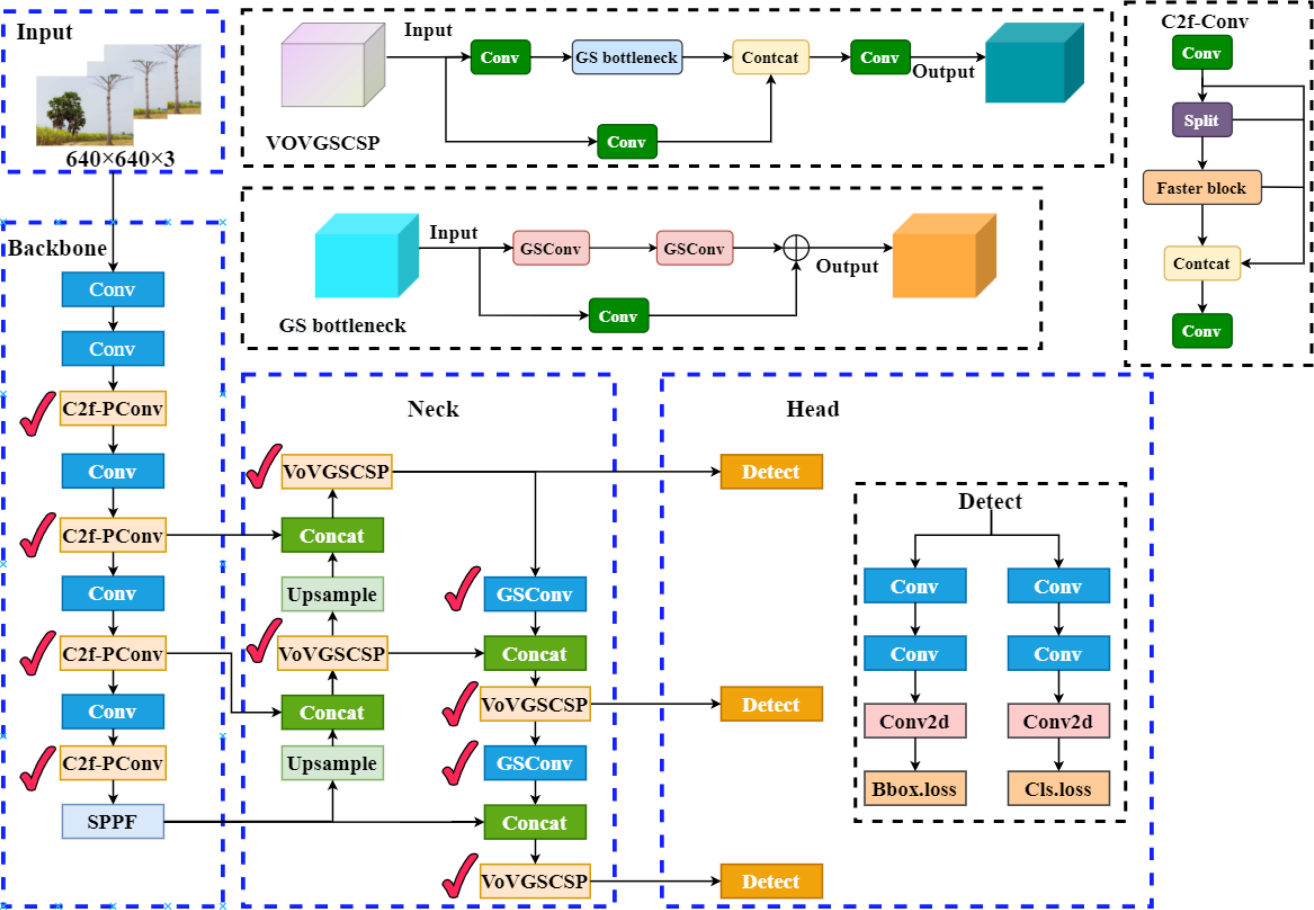
YOLOv8-PSS model structure diagram.

The key improvements of the model presented in this paper include: the incorporation of PConv in the backbone network, which reduces redundant computations and memory accesses while efficiently extracting feature information; the introduction of a Slim-neck in the neck network, replacing the original convolution module with GSConv, and designing a cross-level local network, VoVGSCSP, based on it. This reduces computational complexity and network structure while maintaining sufficient accuracy. Additionally, the original YOLOv8’s CIoU loss function is replaced with the Shape-IoU loss function, enhancing both detection accuracy and convergence speed. The structure of the improved detection model is shown in **Figure 2**, with the marked areas indicating the improvements.

#### 2.3.1 PConv

The C2F module in YOLOv8 was originally designed to extract effective features. However, in certain cases, feature redundancy may still occur, leading to wasted model parameters and computational resources, ultimately affecting the model’s generalization ability. While many lightweight network improvements focus on reducing computation, this often increases the number of memory accesses required during image processing, thereby reducing computational efficiency. This trade-off can not only slow down the model’s overall speed but also degrade its accuracy to some extent. Therefore, this research introduces the partial convolution (PConv) proposed in FasterNet, PConv (Chen et al., 2023) compared to conventional convolutional operations. In partial convolution, regular convolution is applied to only a subset of the input feature map’s channel, while the remaining channels remain unchanged. This enables PConv to more efficiently select the first or last consecutive channels as representatives of the entire feature map for computation when performing consecutive memory accesses. By doing so, it optimizes the use of the device’s computational power, reduces the amount of model computation and memory consumption, avoids performance degradation caused by inter-channel redundancy, and improves the model’s generalization ability. During calculations, simplifying the PConv calculation as $h\times w\times k^{2}\times c_{p}{}^{2}$ , reduces the need for memory accesses with the following expression for memory accesses:


(1) $$h\times w\times k^{2}\times 2c_{p}+k^{2}\times c_{p}^{2}\approx h\times w\times 2c_{p}$$

where *h and w* denote the length and width of the input feature map, *c* denotes the number of input channels, *c_p_* denotes the channels involved in convolution, and *k* is the convolution kernel size.

**Figure 3:**
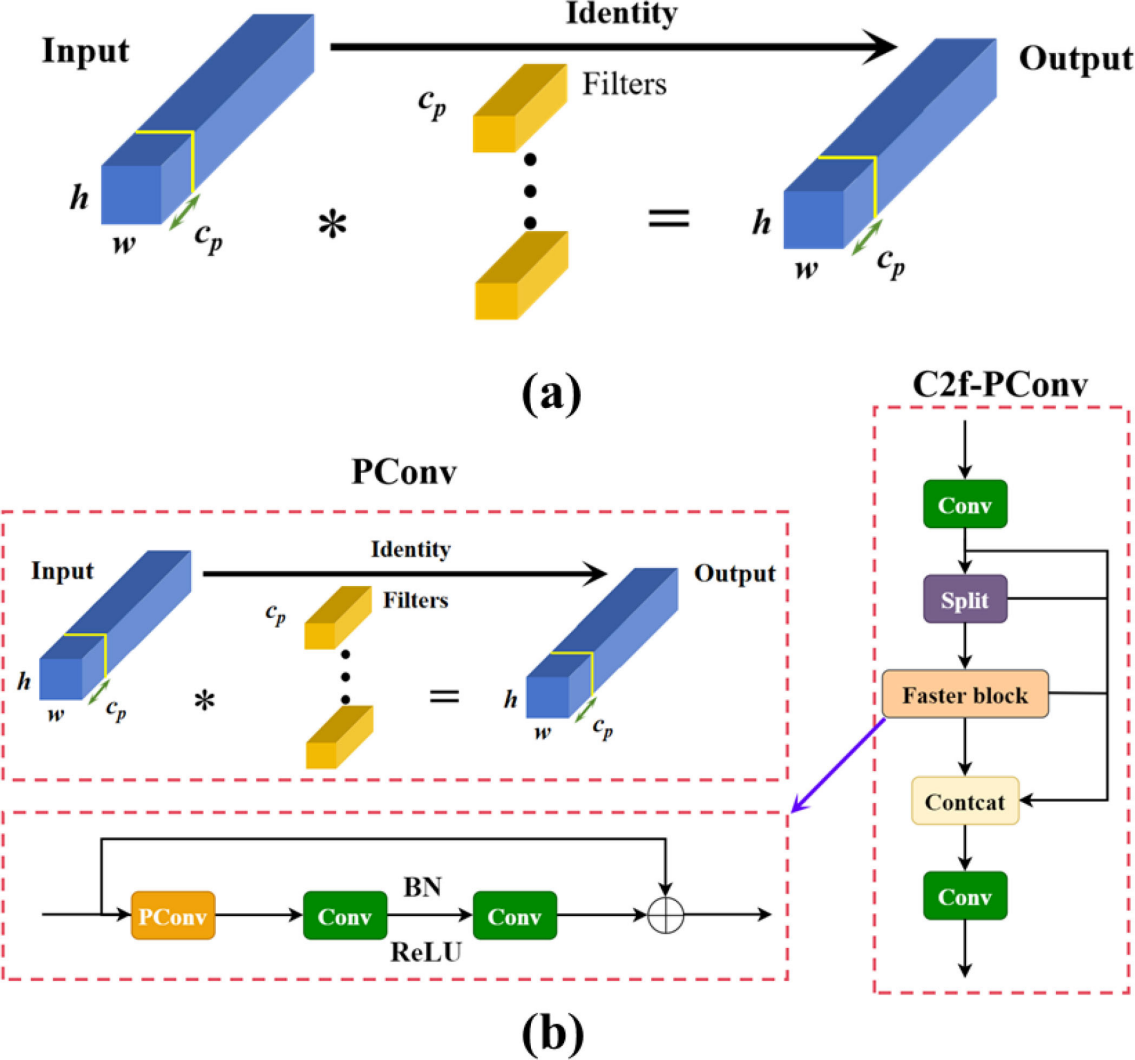
**(A)** Processing of the output feature maps from the regular convolution module; **(B)** Diagram of the structure of the C2f-PConv module; * denotes convolution processing.

In this paper, PConv is used to streamline and improve the C2f module, maintaining the extended feature extraction capabilities of C2f. A Faster Block is introduced to replace the Bottleneck in C2f, further reducing the computational load during the calculation process. The structure of PConv and the modified module is shown in **Figure 3**.

#### 2.3.2 Slim neck

**Figure 4:**
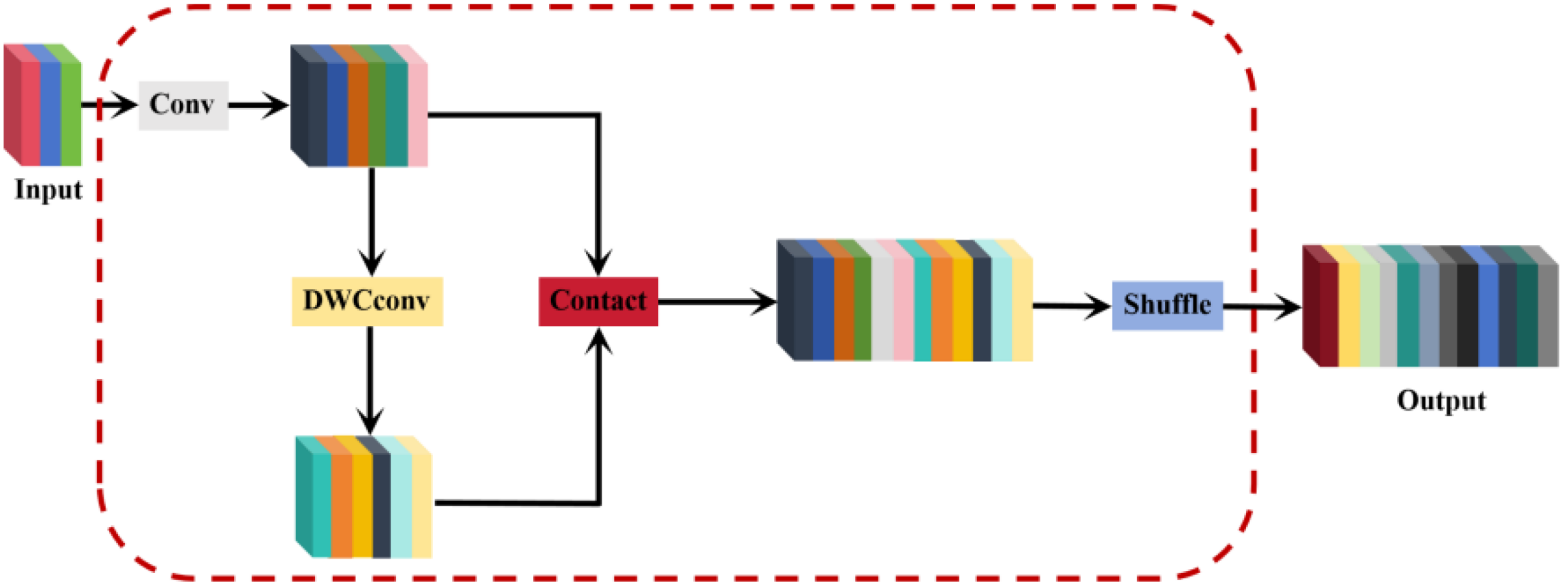
GSConv structure diagram.

In traditional CNNs, spatial information is gradually transformed into channel information, leading to a partial loss of semantic data during spatial compression and channel expansion at each feature map layer. When using standard convolution (SC) for feature extraction, the number of parameters grows with the increasing depth of the network, impacting detection efficiency. Depth-wise separable convolution (DSC) has become popular in many lightweight designs to reduce computation and improve efficiency. DSC minimizes parameters by performing stepwise convolution, which processes channel information separately. However, this approach captures complex spatial information and feature relationships less effectively than SC. Therefore, a balance must be struck between the computational efficiency of DSC and the performance of SC. Li et al (Li et al., 2024). proposed GSConv, a novel approach designed to maintain hidden connections between channels as much as possible while keeping the time complexity low. The core idea of GSConv is to enhance the fusion of feature information between different groups by introducing a shuffle operation that mixes the information generated by the SC operation into each part of the information generated by the DSC. The shuffle operation improves the performance of the model by mixing information from different groups by exchanging local feature information uniformly over different channels. Therefore. In this paper, GSConv is adopted as a lightweight convolution method, replacing both SC and DSC convolutions and integrating the Slim-neck lightweight neck network. This ensures a balance between the detection accuracy and computational efficiency of the network. The structure of GSConv is shown in **Figure 4**. The Slim-Neck module introduces a GS bottleneck and VoVGSCSP lightweight module based on GSConv, to solve the problem of increased computation caused by many C2F modules extracting features in the YOLOv8 neck network as well as solving the problem of difficulty in real-time detection of the deployed devices at runtime.

**Figure 5:**
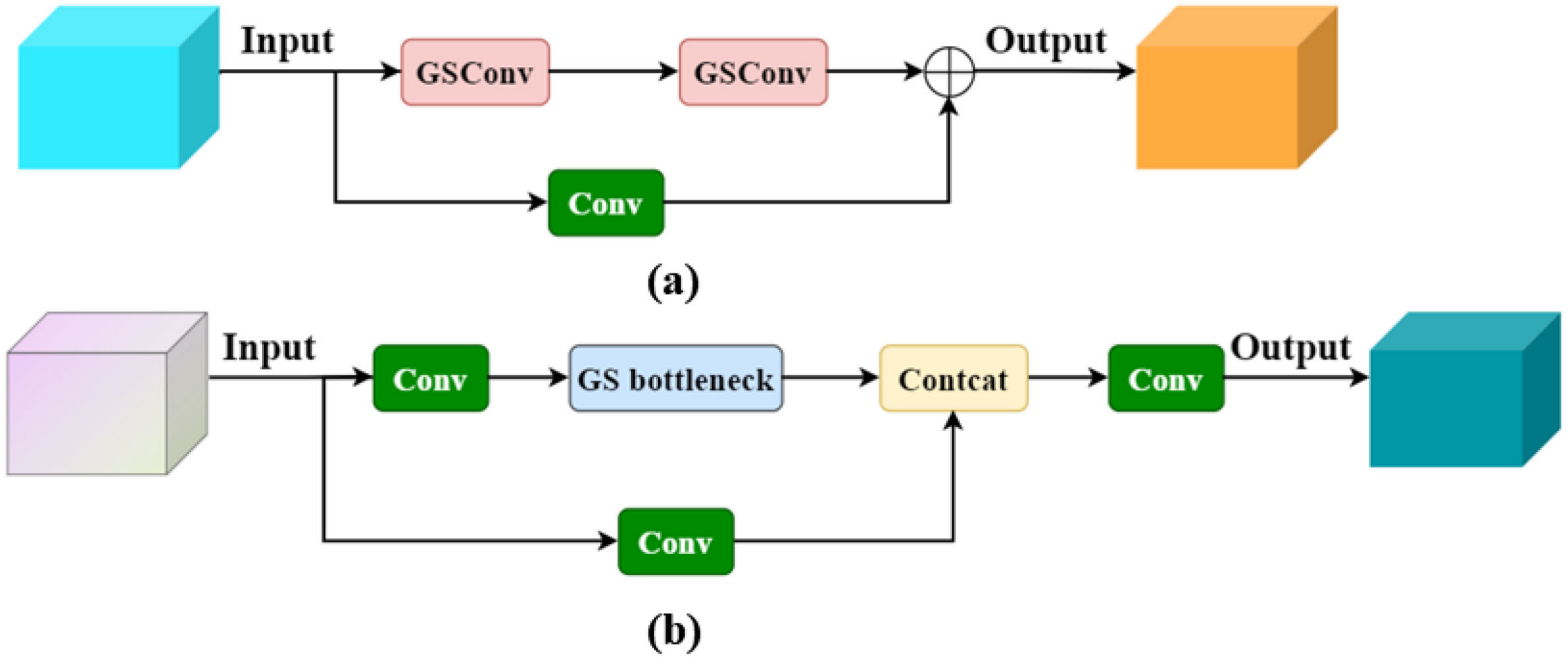
**(A)** Structure of GS bottleneck; **(B)** Structure of VoV-GSCSP.

To enhance the inference speed of the network model while preserving its detection accuracy, the cross-level partial network module VoV-GSCSP was designed to replace the original C3 module, utilizing the GSConv module. This design cleverly integrates GSConv with VoVNet, reducing the number of parameters and computational complexity. The VoV-GSCSP module ensures high computational efficiency while improving the overall network performance and maintaining sufficient accuracy. **Figure 5A** illustrates the structure of the GS bottleneck, and **Figure 5B** shows the structure of the VoV-GSCSP.

#### 2.3.3 Shape-IoU

The loss function serves to quantify the difference or error between the model’s predicted labels and the actual labels. By calculating the loss value, it is clear how well the model performs on a given dataset, i.e., the accuracy of the model prediction. The CIoU loss function used in YOLOv8 only considers the overlapping area of the two bounding boxes but does not consider the target’s size, shape, and positional feature information. In some cases, it may not accurately reflect the actual situation, leading to a decrease in detection accuracy.

**Figure 6:**
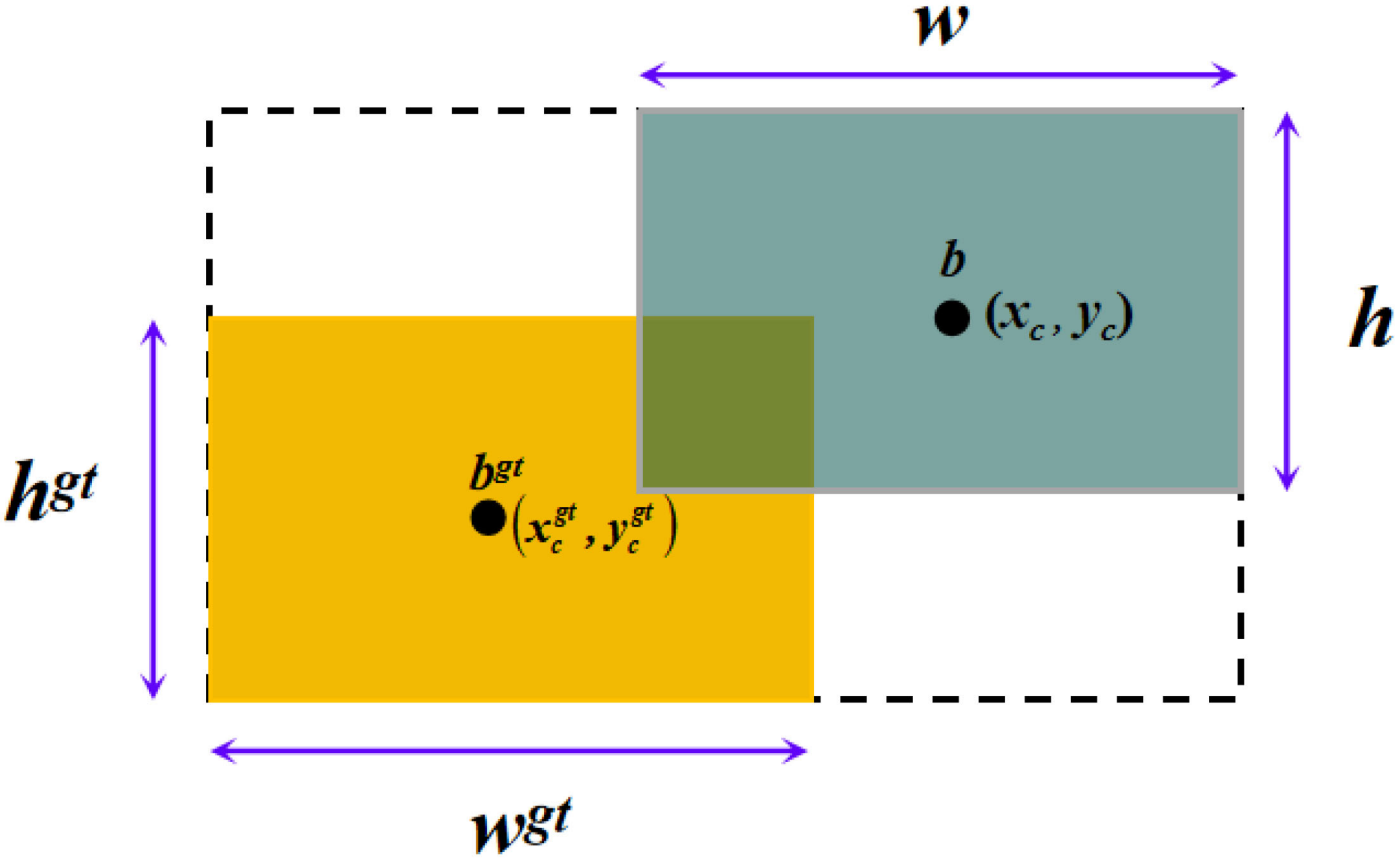
Schematic diagram of the intersection of the true and predicted boxes.


In contrast to CIoU, the Shape-IoU loss function calculates the loss by focusing on the shape and scale of the bounding box, resulting in more precise border regression and enhanced detection accuracy and robustness (Zhang and Zhang, 2023), as shown in **Figure 6**. Therefore, in this paper, Shape-IoU is used to replace CIoU in the original model. This modification not only improves the network’s convergence performance but also enhances its ability to detect obstacles more accurately. The expression for Shape-IoU is shown below.

(2) $$L_{Shape-loU}=1-IoU+disance^{shape}+0.5\;\times \Omega^{shape}$$

(3) $$distance^{shape}=hh\times \frac{{\left(x_{c}-x_{c}^{gt}\right)}^{2}}{c^{2}}+ww\times \frac{{\left(y_{c}-y_{c}^{gt}\right)}^{2}}{c^{2}}$$

(4) $$\Omega^{shape}=\sum_{t=w,h}{\left(1-e^{-\omega_{t}}\right)}^{\theta }$$

(5) $$ww=\frac{2\times {\left(w^{gt}\right)}^{scale}}{{\left(w^{gt}\right)}^{scale}+{\left(h^{gt}\right)}^{scale}}$$

(6) $$hh=\frac{2\times {\left(h^{gt}\right)}^{scale}}{{\left(w^{gt}\right)}^{scale}+{\left(h^{gt}\right)}^{scale}}$$

(7) $$\Omega^{shape}=\sum_{t=w,h}{\left(1-e^{-\omega_{t}}\right)}^{\theta }$$

(8) $$\{\begin{array}{c} \omega_{w}=hh\times \frac{\left|w-w^{gt}\right|}{m\alpha x(w,w^{gt})}\\ \omega_{h}=ww\times \frac{\left|h-h^{gt}\right|}{m\alpha x(h,h^{gt})} \end{array}$$

Where $\Omega^{shape}$ is the shape loss; $w^{gt}$ , $h^{gt}$ denotes the width and height of the real bounding box, respectively; θ is the degree of control attention to shape loss and takes the value of 4.

### 2.4 Visualization of heat maps

**Figure 7:**
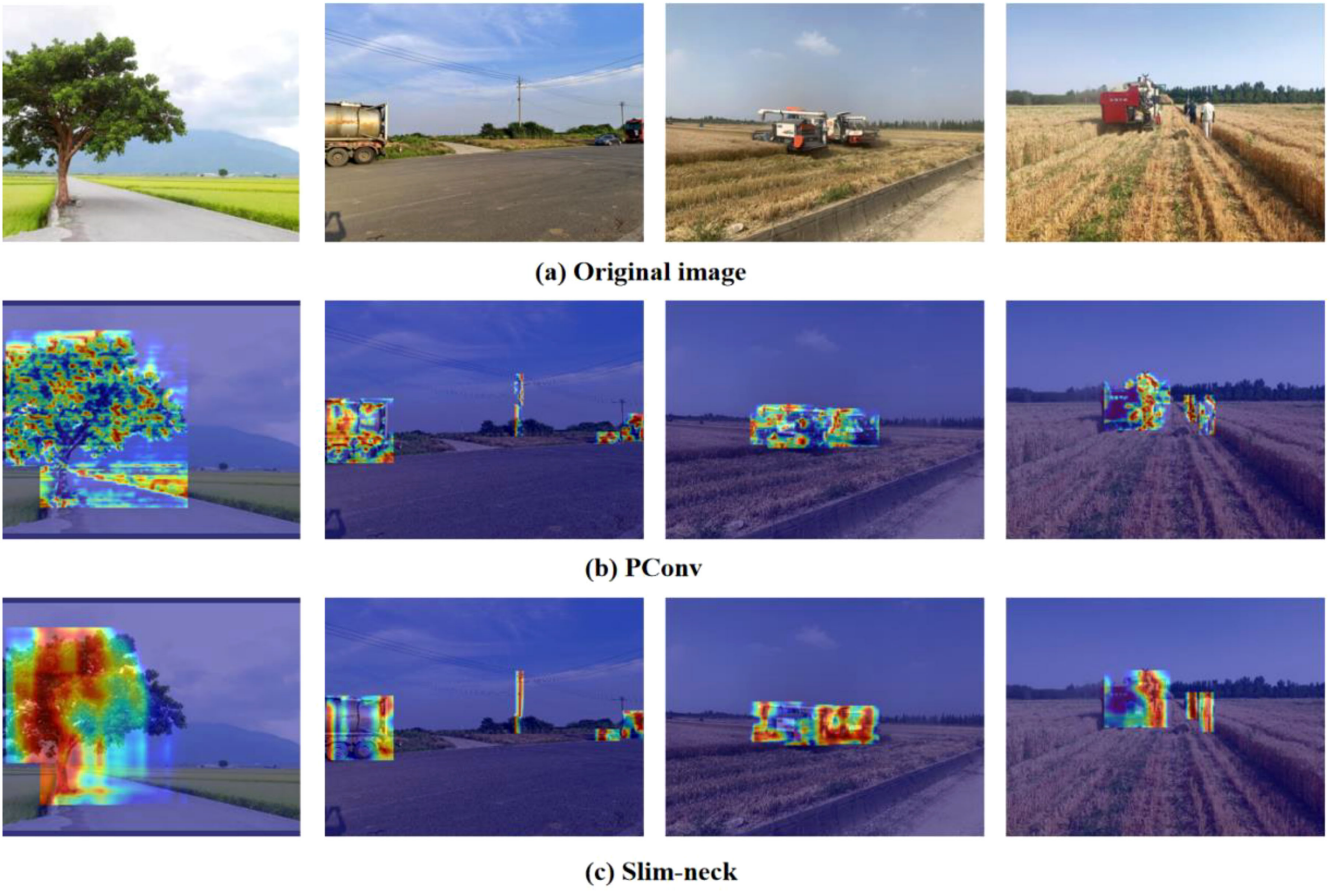
Visualization results of heat maps under different network modules (**(A)** Original image, **(B)** PConv, **(C)** Slim-neck).

Gradient-weighted Class Activation Heatmap (Grad-CAM) can be used to interpret the results predicted by the model by visualizing the heatmap that the model considers most significant. In obstacle detection, Grad-CAM generates heatmaps for the key regions making it evident where the model focuses its attention on the obstacle’s critical information. The basic idea of Grad-CAM (Liu et al., 2024) is to use the gradient of the neural network in the output layer for a specific class to determine which feature maps are the most important for predicting a specific class by calculating the gradient weight of the feature map of the last convectional layer. To showcase the improved detection performance of the model, Grad-CAM is applied to visualize the feature extraction at various layers, with red representing higher weights, where the model pays more attention to the region, and blue represents lower weights, which means that the model pays less attention to the region. To observe the heat maps more clearly, the heat maps are superimposed on top of the original maps. Thus, the different features and the areas on which the model focuses can be seen clearly. As shown in **Figure 7**, the embodiment of the improved model’s focus on features at different stages can be seen, and the experimental results show that the algorithm can better solve the obstacle recognition problem in different scenarios.

### 2.5 Experimental platforms

In this paper, we use our own paired workstation for the training and validation of the deep learning part, the hardware configuration includes: In this study, the training and validation of the deep learning model were conducted on a custom-built workstation. CPU is Intel Core i9 -13900kf, running memory is 32GB, GPU is NVIDIA RTX 4090 graphic card, 1T solid state hard drive; the software is running on the 64-bit operating system of Windows 10 (22H2)All programs are written in python language using the Pytorch1.12 deep learning framework with training acceleration powered by NVIDIA CUDA 11.6 parallel computing driver. Deep learning framework PyTorch 2.1.2, programming platform PyCharm, programming language Python 3.8, CUDA version 11.3, and CUDNN version 7.6 were utilized. These optimizations contribute to improved model accuracy and generalization after applying lightweight techniques.

The training parameters were all adopted from the default hyper-parameters of The input image resolution was set to 640×640 pixels with a batch size of 32, and pre-trained weights were utilized. Stochastic Gradient Descent (SGD) was employed to optimize the network and accelerate the convergence process. The learning rate was set at 0.01, with weight decay coefficients of 0.0005, and a momentum factor of 0.937 A total of 300 rounds of iterations were conducted followed by an optimal results analysis.

### 2.6 Evaluation metrics

This research utilizes standard performance evaluation metrics commonly used in target detection, including precision (P), recall (R), mean average precision (mAP), the number of parameters, floating point operations (FLOPs), model size, and frames per second (FPS) (Zhang et al., 2023). mAP is particularly important for assessing the overall detection performance of the target detection model; a higher mAP value indicates better obstacle detection capability. To evaluate model complexity, three main metrics are considered: the number of parameters, FLOPs, and the model size. P and R are calculated using true positive (TP), false positive (FP), true negative (TN) and false negative (FN). mAP is particularly important for assessing the overall detection performance of the target detection model; a higher mAP value indicates better obstacle detection capability. To evaluate model complexity, three main metrics are considered: the number of parameters, FLOPs, and the model size. FLOPs represent the speed of floating-point operations, which can be used to measure the complexity of a model. Parameters represent the computational memory resources consumed by the model. The formulas are as follows:


(9) $$P=\frac{TP}{TP+FP}$$

(10) $$R=\frac{TP}{TP+FN}$$

(11) $$AP=\int_{0}^{1}P(R)dR$$

(12) $$mAP=\frac{\sum_{n=1}^{N}AP_{n}}{N}$$

(13) $$Parameters=r^{2}\times a\times \nu +\nu$$

(14) $$FLOP_{S}=2\times H\times W\times C_{out}\times (C_{in}\times K^{2}+1)$$

where *N* is the number of categories. where *a* is the input size, *r* is the size of the convolution kernel, *v* is the output size, *H ×W* is the size of the output feature map, *C_in_* is the input channel, *K* is the kernel size, and *C_out_* is the output channel.

### 2.7 Detection and positioning system design

In this study, the OAK-D-Pro camera from Luxonis is selected for the study, featuring two infrared cameras, one RGB camera, and an infrared laser dot-matrix transmitter. The depth measurement principle mainly relies on the parallax generated by the infrared cameras in the imaging plane to calculate the distance to the object. Firstly, the RGB and depth images of the front view are captured by the two IR cameras and the RGB camera. The improved YOLOv8 model is then applied to detect obstacles within the RGB image. The parameters of the OAK-D-Pro camera are shown in **Table 2**.

**Table 2:** OAK-D-Pro Parameters.

Parameters of OAK-D-Pro	RGB camera	Infrared camera
Resolution	4032×3040	1280× 800
Baseline length	/	75 mm
Camera focal length	4.81	2.35
FOV	81°D/69°H/55°V	81°D/72°H/49°V
Size	97 × 29.5 × 22.9 mm	
Power	2 ~ 5.5 W	

To obtain the precise position point of the obstacle in real space, it is necessary to go through the conversion from the pixel coordinate system, and image coordinate system to the camera coordinate system, and finally transform the 2-D image coordinates into 3-D spatial coordinates. In the obstacle positioning system, the camera is used as the origin of the coordinate system to locate the obstacle, and after identifying the pixel coordinates of the obstacle, the 3-D coordinates of the obstacle in the camera coordinate system combined with the depth information are used to compute the absolute coordinates in the world coordinate system after the final coordinate transformation, to realize the detection and positioning.

**Figure 8:**
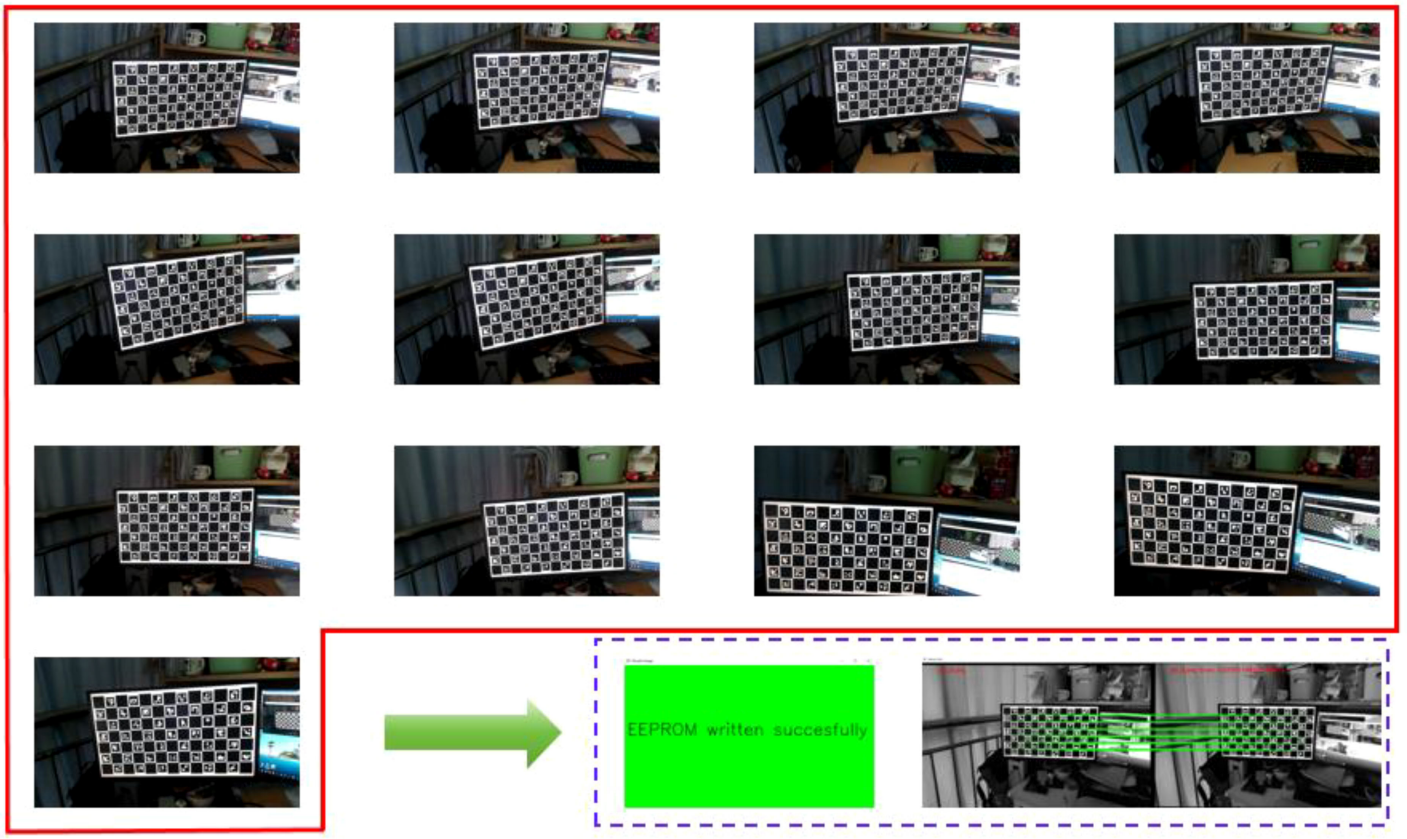
Camera calibration diagram.

To ensure the accuracy of camera positioning, OAK-D-Pro needs to be calibrated by displaying a checkerboard calibration image on a 24 inch flat display, capturing a total of 13 multi border positions for calibration. After capturing images of all multi border positions, the calibration image processing step will begin. Upon successful completion, a green background interface will pop up at the end. The checkerboard positions at different calibration angles and the interface after successful calibration are shown in **Figure 8**.

After calibration is completed, generate the cam_chain.yaml file, which contains the internal and external parameters of the camera after calibration, as shown in **Table 3**.

**Table 3:** Calibration parameters.

Calibration parameters	center camera parameters	Right camera parameters
Radial distortion parameter/*k_1_*	0.0281	0.0129
Radial distortion parameter/*k_2_*	-0.0682	-0.0458
Tangential distortion coefficient/*p_1_*	-0.0013	-0.0014
Tangential distortion coefficient/*p_2_*	0.0024	0.0047
U-axis scale factor/*fx*	806.2	797.1
V-axis scale factor/*fy*	665.4	642.6
Rotation matrix/*R*	$\left[\begin{array}{ccc} 0.9999 & 0.0027 & 0.014\\ -0.0023 & 0.9999 & 0.0031\\ -0.0056 & -0.0029 & 0.9999 \end{array}\right]$	
Offset matrix/*T*	$\left[\begin{array}{c} 3.7351\\ -0.1476\\ 0.0041 \end{array}\right]$	

**Figure 9:**
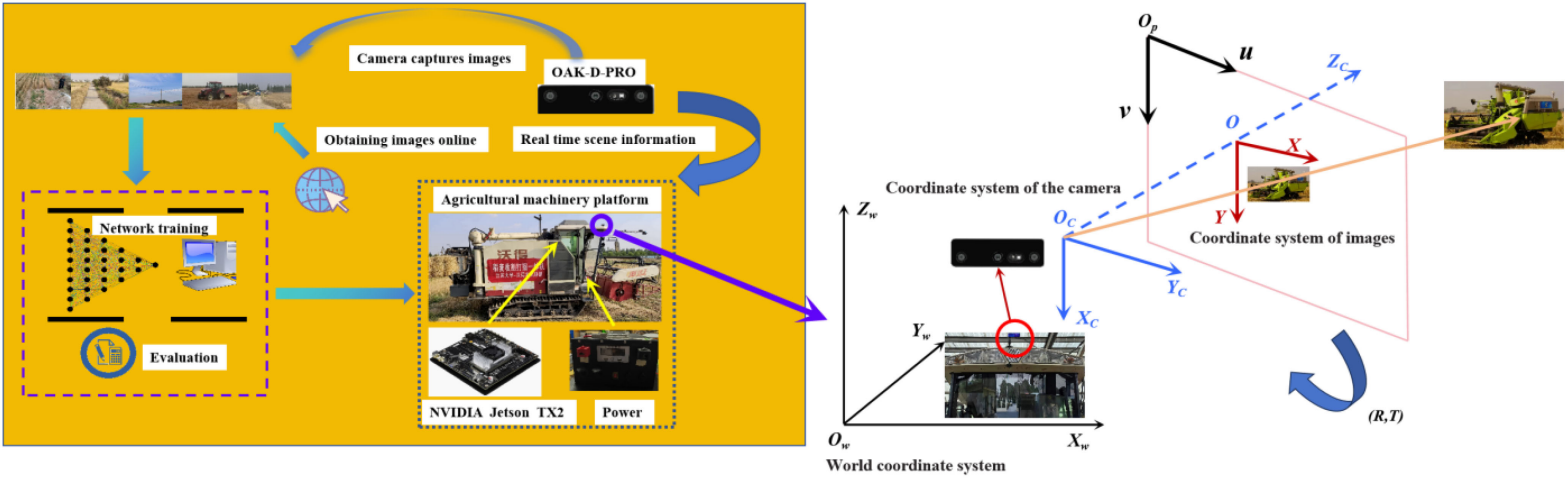
Overall framework diagram.

To simulate real-world conditions, the OAK-D-Pro camera was mounted on a tracked rice and wheat harvester capturing the video information in the actual field environment. The improved YOLOv8 model was then deployed on the NVIDIA Jetson TX2 to detect and identify the obstacle targets, which still rely on the manually driving operation in the process. The overall framework of the system is shown in **Figure 9**.

## 3 Discussion and analysis

### 3.1 Ablation experiment

The ablation test results, using the same obstacle dataset and training environment, are presented in **Table 4**. The model incorporating the PConv module is referred to as YOLOv8_P, and the model utilizing the Slim-neck module is designated as YOLOv8_S, with other models named similarly based on their improvements. As observed in **Table 4**, replacing the C2f module in YOLOv8 with the C2f-PConv in Test 1 resulted in the YOLOv8_P model. While there was a slight decrease in precision, recall, and average precision compared to the original YOLOv8, the model’s weight was reduced by 36.2%. Additionally, the number of parameters and computational load were reduced to 67.3% and 79.3%, respectively, of the original model. The main reason for the decrease in the precision rate and other values is that only some channels are involved in the convolution operation of PConv, which has a certain feature extraction effect, but at the same time reduced the amount of computation and size of the model, it also loses some of the feature information in the remaining channels, leading to a reduction in the precision of the training. It is worth noting that the results of experiment 2 show that although there is a slight decrease in the model detection performance after adding PConv, the magnitude of the decrease is within an acceptable range. By sacrificing a small part of the accuracy, the computational complexity and complexity of the model are greatly reduced, which is more conducive to the deployment of the model on the platform in the future. Experiment 3 based on experiment 1, the neck network was redesigned using the Slim-neck network. At that time, the precision, recall, and average accuracy of the model were 84.3%, 88.1%, and 89.5%, compared with the baseline model, the precision and detection speed of the redesigned model were slightly improved. The number of participants, the amount of computation, and the size of the model were 51.9% of that of the original model, respectively, 55.3%, and 48.3%, indicating that the Slim-neck lightweight network used in this research can meet the demand of real-time detection in terms of model detection accuracy. The main reason for the improved performance is the replacement of traditional convolution and the C3 module in the original model with GSConv and VoV-GSCSP modules. This modification allows the structure of the Slim-neck network to preserve the important channel connections in the neck structure. The GSConv module can effectively process channel feature information to avoid redundant information from being compressed repeatedly. As a result, the model’s feature extraction capability and detection speed are enhanced without sacrificing accuracy. Experiment 4 is based on experiment 2, and the original model neck network and backbone network are simultaneously improved by light-weighting, which greatly reduces the complexity and computation amount of the model while maintaining a slight decrease in model accuracy. The size of the improved model is 4.7 MB, the number of parameters is 6.0 G, the number of model parameters is 2.3 × 106 M, the precision, recall, and average accuracy are 83.9%, 85.8% and 89.4%, the mAP decreases by 0.5%, and the detection speed is 113 frames/s. The number of parameters, the amount of computation, and the size of the improved model are about half of the previous ones, and the detection speed is increased by 14%. Experiment 5 based on Experiment 4; the original loss function is further replaced by Shape-IoU. The model has improved precision, recall, and average precision compared with the previous one. The complexity of the model remains unchanged, which indicates that Shape-IoU can improve the fitting effect of the model and further improve the detection accuracy of the model.

**Table 4:** Improved model ablation test results.

Number	Model	PConv	Slim-neck	Shape-IoU	P%	R/%	mAP/%	Size/MB	Parameters/×10^6^M	Computation/G	FPS
1	YOLOv8	×	×	×	83.5	86.7	88.9	11.6	5.2	12.3	92
2	YOLOv8_P	√	×	×	83.2	86.4	88.3	7.4	3.5	7.5	107
3	YOLOv8_S	×	√	×	84.3	88.1	89.5	5.6	2.7	6.8	101
4	YOLOv8_PS	√	√	×	83.9	85.8	89.4	4.7	2.3	6.0	113
5	YOLOv8_PSS	√	√	√	85.3	88.4	90.6	4.7	2.3	6.0	111

√ represents the use of the module, and x represents that the module has not been used.

The detection performance across different obstacle types in complex scenes is a key factor affecting the accuracy of the detection task. The detection results of the improved model for different obstacle types are shown in **Table 5**. From **Table 5**, the model’s detection effect for dynamic obstacles is better than that for static ones, the model has the best detection effect for pedestrians, with P, R, and AP of 88.5%, 90.2%, and 92.3%, respectively, and the model has the worst detection effect for utility poles, with P, R and AP of 81.3%, 86.7% and 88.4%, respectively. There are some differences in the model’s handling of different obstacles, and the known static obstacles detected during travelling can be labelled in subsequent obstacle avoidance studies to further enhance the safety of travelling.

**Table 5:** Information on different obstacle detection accuracy.

Type	P(%)	R(%)	mAP(%)
Person	88.5	90.2	92.3
Tree	82.1	86.2	89.6
Poles	81.3	86.7	88.4
Vehicle	86.7	88.6	90.9
Agm	87.9	90.3	91.8
Average value	85.3	88.4	90.6

### 3.2 Comparison of results from different detection models

To further verify the actual performance of the improved model proposed in this paper, this paper compares the improved model with the mainstream two-stage target detection algorithm Faster R-CNN and the one-stage target detection algorithms SSD, YOLOv3-tiny and YOLOv5 models under the same conditions of test parameters and configuration environments, and the comparison results are shown in **Table 6**.

**Table 6:** Comparison of detection results of different models.

References	Model	P/%	R%	mAP/%	Size/MB	Parameters/×10^6^M	Computation/G	FPS
(Ren et al., 2016)	Faster RCNN	74.3	91.9	43.7	108	86.9	129.8	41
(Hui et al., 2019)	SSD	84.6	80.3	80.1	93.1	31.4	58.7	65
(Bin et al., 2021)	YOLOv3-tiny	82.8	81.2	79.5	24.5	20.7	46.3	77
(Su et al., 2022)	YOLOv5	80.7	84.6	83.9	13.2	9.0	15.2	86
	YOLOv8_PSS	85.3	88.4	90.6	4.7	2.3	6.0	111

As demonstrated in **Table 6**, the enhanced YOLOv8 model outperforms Faster R-CNN, SSD, YOLOv3-tiny, and YOLOv5 by 11%, 0.7%, 2.5%, and 4.6%, respectively. Furthermore, the mean Average Precision (mAP) increases by 46.9%, 10.5%, 11.1%, and 6.7% when compared to these models. The improved YOLOv8 model also exhibits a significant reduction in model size, number of parameters, and computational load. Although the recall rate of Faster R-CNN is higher than that of YOLOv8-PSS during obstacle detection, the other performance metrics of YOLOv8-PSS are superior. Despite being a two-stage target detection model, Faster R-CNN’s substantial parameter count renders it unsuitable for lightweight deployment requirements. The SSD algorithm demonstrates higher precision and accuracy compared to Faster R-CNN. Furthermore, YOLOv3-tiny and YOLOv5 have a greater number of parameters and higher floating-point computation requirements than the proposed improved method, and they also exhibit lower average accuracy, which is not conducive to the lightweight design of the network. The model in this research is improved based on YOLOV8, compared with other mainstream models, the improved algorithm proposed in this research achieves the lowest number of parameters and the computational requirements, and shows the highest detection accuracy, which meets the lightweight network at the same time, and meets the deployment requirements of the obstacle detection effect. It is worth noting that the YOLOv8-PSS model proposed in this article has certain limitations in detecting objects with similar shapes, and there is still room for improvement in the detection performance of the model. The main reason may be that many obstacles in the self-built dataset in this article were collected in different scenarios, and in order to run the model on mobile platforms, the focus of this article is more on the lightweight research of the model. Therefore, in the subsequent research process, in order to further improve the detection performance of the model, more datasets will be obtained for training, and attention mechanisms will be added to the model to enhance detection performance. In summary, this article introduces a lightweight YOLOv8-PSS model for obstacle detection in agricultural machinery during operation, which has high efficiency and accuracy. This is of great significance for the research of obstacle avoidance in unmanned agricultural machinery.

**Figure 10:**
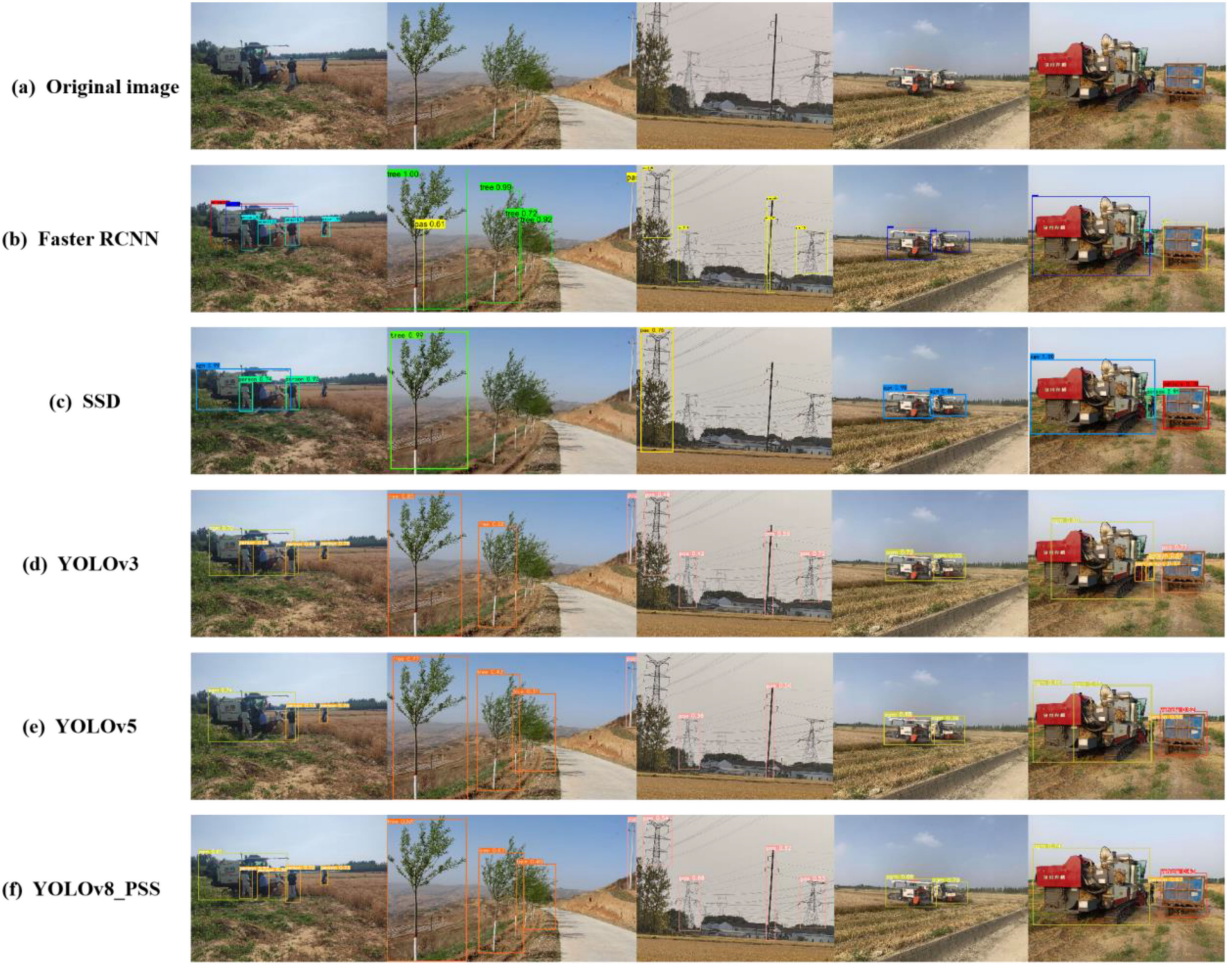
Comparison of the effectiveness of different detection models (**(A)** original image, **(B)** Faster RCNN, **(C)** SSD, **(D)** YOLOv3, **(D)** YOLOv5, **(F)** YOLOv8_PSS).

To check whether the model can recognize the obstacle information, various scene input models were tested for obstacle detection, and the detection results are shown in **Figure 10** From the figure, Faster R-CNN and YOLOV8_PSS performed better for the detection of obstacle information, SSD, YOLOv3-tiny and YOLOv5 have some degree of omission when recognizing different scenes, including people, poles, and trees. Notably, YOLOv5 encountered issues with misidentification, mistakenly recognizing a single farm machine as two separate entities. Although the algorithm proposed in this paper demonstrates strong performance in obstacle detection, it still faces challenges with tree detection. This is mainly because when the tree overlaps or occludes the phenomenon. This overlap complicates the model’s ability to extract distinguishing features, ultimately leading to instances of missed detection.

### 3.3 Comparison of different modules

To verify the effectiveness of the PConv and Slim neck modules used, this paper uses an improved YOLOv8 object detection network as the base network. By replacing the mainstream lightweight backbone network and neck network, while maintaining the same parameters except for the backbone or neck network, the effects of different modules on target training are compared. The specific results are shown in **Tables 7** and **8**. According to **Table 1**, PConv has better advantages in mAP compared to ShuffleNetV2, MobileNetV2, and GhostNet backbone networks, with improvements of 9.2%, 6.4%, and 2.7%, respectively. Although MobileNetV2 has lower computational complexity and model size than PConv, its obstacle calibration performance is poor and it is not suitable for practical deployment situations. According to **Table 3**, the Slim neck module has a certain improvement effect compared to other neck network improvement methods, with mAP increased by 10.3%, 10.9%, and 4.1%, respectively. Therefore, the use of Slim neck for lightweight improvement of the neck network has significantly improved the overall detection performance of the model compared to the original network, which can meet real-time detection requirements and is more suitable for mobile deployment of later models.

**Table 7:** Comparison of different lightweight backbone networks.

Backbone	P/%	R/%	mAP/%	Size	Computation/G
MobileNetV2	83.4	73.9	81.4	4.5	5.8
ShuffleNetV2	82.1	76.6	84.2	6.9	8.1
GhostNet	82.3	83.6	87.9	6.7	8.4
PConv	85.3	88.4	90.6	4.7	6.0

**Table 8:** Comparison of improvements in different neck networks.

Backbone	P/%	R/%	mAP/%	Size	Computation/G
Bifpn	77.7	82.1	80.3	6.3	8.2
RepGFPN	79.4	83.5	79.7	5.7	7.6
GD-YOLO	80.2	81.9	86.5	5.8	6.3
Slim neck	85.3	88.4	90.6	4.7	6.0

To further assess the effectiveness of the Shape-IoU bounding box loss function, we compared its convergence with that of the SIoU, CIoU, EIoU, and WIoU loss functions in enhancing the network’s performance. The improved model comprises 2.3 × 10^6^ parameters, with a computational load of 6.0 G and a model size of 4.7 MB when different bounding box loss functions are employed. The results indicate that substituting these loss functions does not impact the model’s complexity. From **Table 9**, changing the bounding box loss function affects the precision, accuracy, and recall of the model to a certain extent. The recall of the improved model using Shape-IoU is 88.4%, which is 2.8%, 2.7%, 3.3%, and 4.6% higher than that of CIoU, SIoU, EIoU, and WIoU, respectively. Additionally, the mAP for the shape-IoU is 90.6%, which is 1.2%, 2.1%, 1.9%, and 0.7% higher than the corresponding values of other models. Although Shape-IoU precision is 0.9, 0.1, and 0.2% lower than SIoU, EIoU and WIoU, respectively, the optimal equilibrium between recall and mean average precision is achieved.

**Table 9:** Performance comparison of different bounding box loss functions.

Loss function	P/%	R/%	mAP/%
CIoU	83.9	85.6	89.4
SIoU	86.2	85.7	88.5
EIoU	85.4	85.1	88.7
WIoU	85.5	83.8	89.9
Shape-IoU	85.3	88.4	90.6

**Figure 11:**
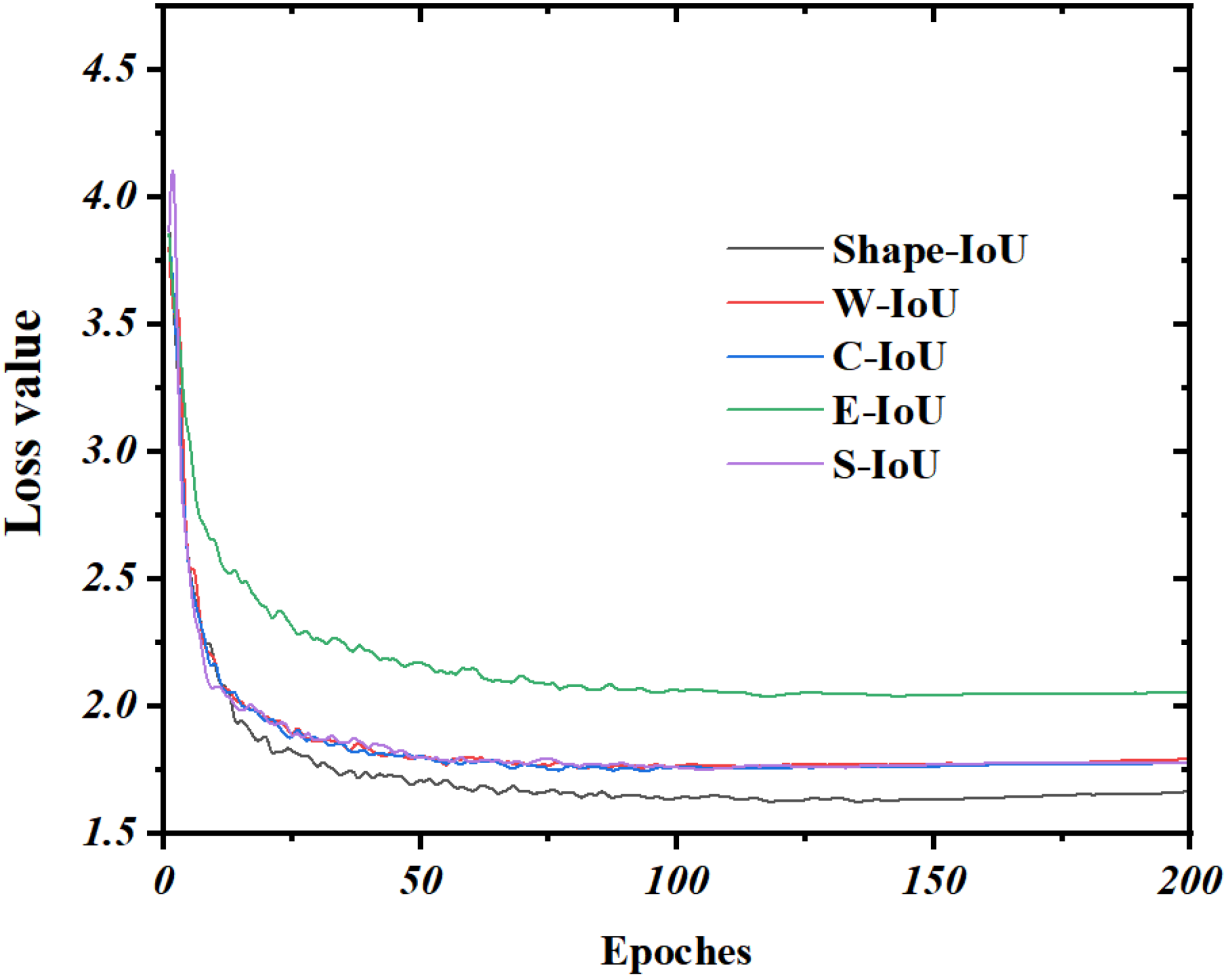
Iterative variation curves of the bounding box loss function.

As shown in **Figure 11**, it is the convergence of YOLOv8 lightweight improved model respectively using different bounding box loss functions during training. As depicted, the loss values for the various bounding box loss functions begin to converge after 50 iterations on the validation set. Notably, the regression optimization of the bounding box using the EIoU loss function proves inefficient for datasets with complex scenarios, resulting in the highest bounding box loss value and the slowest convergence speed. In contrast, the SIoU, CIoU, WIoU, and Shape-IoU loss functions converge at a similar rate, achieving significantly lower convergence values compared to EIoU. Among these, the Shape-IoU loss function exhibits a smaller loss value upon convergence, indicating reduced susceptibility to overfitting and stronger generalization ability. Therefore, the Shape-IoU loss function demonstrates the best overall performance in this study.

### 3.4 Model positioning accuracy experiments

To further verify the positioning accuracy of the model in real scenarios, the YOLOv8_PSS model training file was converted into a blob file for compilation operation. Testing was conducted on June 12, 2024, at Rungo Farm Co. in Jingkou District, Zhenjiang City, Jiangsu Province, China. The obstacle detection and positioning distances ranged from 2 to 15 meters. Depth distance measurements were utilized for evaluation and analysis, with the DM120 laser rangefinder produced by DELIXI employed to detect obstacles (measurement accuracy of ± 2 mm and a measurement range of 120 m). The measured results were subsequently analyzed, and the experimental findings are presented in **Table 10**, where Z represents the measurements from the camera and Zd corresponds to the measurements from the laser rangefinder.

**Table 10:** Obstacle positioning accuracy test results.

Number	Tree			Person			Pole			Vehicle			Agm		
	Z/m	Z_d_/m	E/%	Z/m	Z_d_/m	E/%	Z/m	Z_d_/m	E/%	Z/m	Z_d_/m	E/%	Z/m	Z_d_/m	E/%
1	2.42	2.43	0.41	2.66	2.64	0.75	2.47	2.48	0.40	2.48	2.49	0.40	2.86	2.88	0.42
2	3.25	3.28	0.91	3.48	3.52	1.13	3.87	3.83	1.04	3.69	3.72	0.81	3.88	3.92	1.02
3	4.37	4.31	1.39	4.23	4.29	0.93	4.59	4.65	1.29	4.63	4.55	1.76	4.54	4.59	1.09
4	5.41	5.52	1.99	5.48	5.57	1.62	5.09	5.21	2.30	5.81	5.72	1.57	5.34	5.23	2.10
5	6.59	6.73	2.08	6.47	6.65	2.71	6.24	6.03	3.48	6.87	7.03	2.26	6.15	6.34	2.99
6	7.39	7.56	2.25	7.09	7.26	2.34	7.21	7.37	2.17	7.35	7.21	1.94	7.12	7.27	2.06
7	8.23	8.48	2.95	8.87	9.11	2.63	8.56	8.80	2.72	8.52	8.76	2.74	8.96	9.23	2.93
8	9.51	9.78	2.76	9.85	10.15	2.95	9.38	9.66	2.89	9.60	9.91	3.13	9.76	10.12	3.56
9	10.63	10.98	3.19	10.01	10.36	3.39	10.74	11.09	3.15	10.61	10.92	2.84	10.56	10.84	2.58
10	11.02	11.42	3.50	11.25	11.66	3.52	11.71	12.13	3.46	11.03	11.46	3.75	11.82	12.21	3.19
11	12.10	12.55	3.59	12.29	12.74	3.53	12.82	13.28	3.46	12.37	12.81	3.43	12.39	12.84	3.50
12	13.97	14.53	3.85	13.60	14.11	3.61	13.96	14.45	3.39	13.33	13.83	3.62	13.14	13.65	3.73
13	14.77	15.39	4.03	14.39	15.04	4.32	14.94	15.62	4.35	14.86	15.54	4.38	14.68	15.31	4.11
14	15.05	15.75	4.44	15.42	16.12	4.34	15.10	15.77	4.25	15.92	16.63	4.27	15.33	15.99	4.13
Average			2.67			2.69			2.73			2.64			2.67
Maximum			4.44			4.34			4.35			4.27			4.13

**Figure 12:**
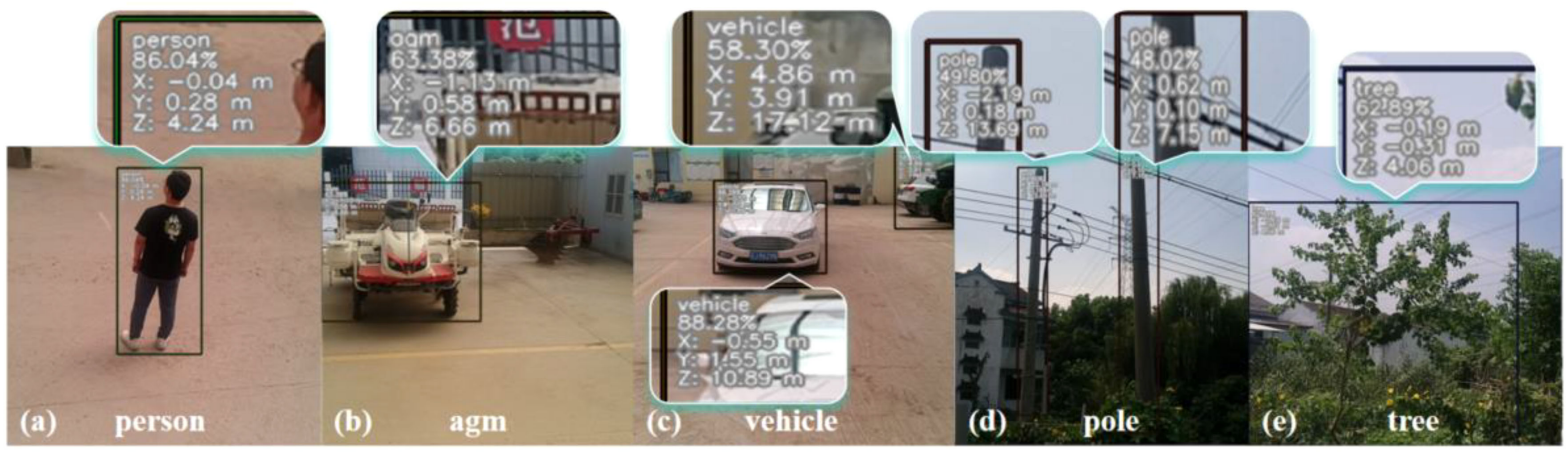
Visualization of the results of field detection and positioning (**(A)** person, **(B)** agm, **(C)** vehicle, **(D)** pole, **(E)** tree).

The test results show that within a distance range of 2 to 15 m, the maximum average relative error of the measured distance is 2.73% while the maximum relative error of the measured distance is 4.44%, respectively. Meanwhile, from **Table 10**, the measurement accuracy for obstacles at middle and near distances is relatively high. However, with the increase of the distance between the obstacles and the camera, the measurement error also increases gradually. This is mainly due to the low maximum resolution of the camera itself during the acquisition, which makes the feature information collected in the collection of information on the objects at a distance is not rich enough. Additionally, outdoor lighting conditions contribute to background noise in the captured images, further affecting positioning accuracy. Positioning accuracy, as can be seen from the table, the overall accuracy of positioning during the test meets the needs of real-time detection. The results of on-site detection are shown in Figure Before testing, the depth camera is mounted on top of the agricultural machine. During operation, the depth camera identifies and detects objects in its field of view, providing their corresponding category, confidence level, and 3D spatial coordinates. As shown in **Figure 12A**, the detected object is identified as a person, with a confidence level of 0.86 and spatial 3D coordinates of (-0.04, -0.28, 4.24) (**Figures 12A–E**). During testing, the detection targets including people, farm machinery, vehicles, utility poles, and trees were 44, 33, 36, 37, and 42, respectively. The number of successfully detected targets was 44, 31, 35 and 38, with the success rates of 100%, 93.9%, 97.2%, 94.5%, and 90.4%, respectively, and the overall success rate of detection was 95.2%.

### 3.5 Research contribution to phytoprotection

Plants are often affected by pests, diseases and factors such as drought and salinity during their complex and varied growth process. To ensure their survival and fulfill environmental protection requirements, it is essential to investigate and develop effective pest and weed control strategies, as well as plant protection technologies, to achieve optimal pest management (ZFrische et al., 2018). With the increasing demand for mechanization in agricultural production, the role of agricultural machinery has gradually shifted from manual to autonomous operation. The use of unmanned agricultural machinery for plant protection has become increasingly widespread. However, due to the complexity and diversity of the unstructured farmland environment, the autonomous operation task of plant protection by unmanned agricultural vehicles require additional attention on obstacle avoidance, in addition to the challenge of achieving full-coverage path planning. Farmland obstacles are not only diverse but also widely distributed, posing a significant threat to the traveling safety of unmanned agricultural vehicles. After detecting obstacles using the YOLOv8-PSS model proposed in this paper, the unmanned farm machine can adjust its travelling path based on the detection results to avoid damaging crops or equipment. For example, an unmanned sprayer used for plant protection can adjust its route according to the location of the obstacle, thus ensuring precise operation and preventing conflicts with crops or other agricultural machinery. The YOLOv8-PSS model is ideal for unmanned agricultural platforms with high efficiency, real-time processing and improved precision in farmland obstacle identification. By accurately identifying obstacles the model enables agricultural equipment to perform precise obstacle avoidance, optimize pesticide application, and enhance crop protection. This improves the intelligence of plant protection machinery and addresses challenges such as high operational precision requirements, complex operating environments, and safety risks associated with unmanned plant protection systems.

## 4 Conclusions

In this research work, to enable rapid obstacle detection during the autonomous navigation of unmanned agricultural machines in complex environments, an obstacle recognition method based on an improved YOLOv8 model is proposed. This approach offers a novel solution for obstacle detection and positioning in agricultural machinery and holds significant theoretical and practical implications for advancing intelligent unmanned agricultural systems. The key conclusions of this study are as follows:

1. Through the construction of farmland obstacle datasets, the YOLOv8_PSS obstacle detection model is proposed. The C2f-PConv module is added to the backbone to reduce network size, while the original neck is replaced by the lightweight Slim-Neck with GSConv and the VoVGSCSP module, cutting model parameters, computation, and size to 55.8%, 51.2%, and 59.5% of the original. Shape-IoU loss is also introduced to stabilize training, reduce regression and shape deviation, and improve convergence speed. The final model achieves 85.3% accuracy, 88.4% recall, and 90.6% mAP, balancing speed and precision better than Faster R-CNN, SSD, YOLOv3-tiny, and YOLOv5, making it ideal for mobile deployment

2. In the obstacle positioning accuracy test, the maximum average error and maximum error for the distance between five types of obstacles and the camera, measured within a range of 2 to 15 meters, were 2.73% and 4.44%, respectively. In the detection test, the model achieved a combined detection rate of 95.2% for obstacles. These results demonstrate the model’s excellent performance in both positioning accuracy and obstacle detection, fulfilling the requirements for precise detection and localization of common obstacles in practical applications. Furthermore, this study sets a benchmark for obstacle detection and localization in unmanned agricultural vehicle environments, offering valuable insights for technological innovation and advancements in related fields. In practical applications, the improved model experiences some degree of obstacle detection failure, particularly in environments with strong or backlighting, where obstacles may blend with the background due to insufficient contrast. Additionally, the model demonstrates lower success rates in recognizing static obstacles compared to dynamic ones. To address these issues, the dataset used in this study primarily consists of images captured during moderate daylight conditions. Future research will aim to enhance the model by expanding the dataset with more diverse samples and further optimizing the model’s architecture.

3. In this study, obstacle detection relied solely on a single stereo camera, which has a limited field of view. The detection system has certain limitations and can only obtain obstacle information on the front side of the agricultural unmanned operation platform. However, in actual road environments, many dynamic obstacles may move towards agricultural machinery from the side. In subsequent research, it is necessary to combine other sensors to detect obstacles, in order to improve the robustness and comprehensiveness of the perception system as much as possible. Different speed ranges and behavioral orientations should be matched for different obstacles, and obstacle avoidance strategies corresponding to different obstacles should be studied to increase the universality of the obstacle avoidance system. These improvements will strengthen obstacle detection in real-world scenarios, especially for dynamic obstacles. This will enhance safety during obstacle avoidance maneuvers, ensuring safer navigation.

## Data Availability

The datasets presented in this article are not readily available because the data is confidential. Any enquires can be directed to the corresponding author.

## References

[B1] Bin C. Man Z. Hongzhen X. Han L. Yanxin Y. (2021). Farmland Obstacle Detection in Panoramic Image Based on Improved YOLO v3 tiny. Trans. Chin. Soc. Agric. Machinery (Transactions CSAM) 52, 58–65. doi: 10.6041/j.issn.1000-1298.2021.S0.008

[B2] Chen J. S-h K. He H. Zhuo W. Wen S. Lee C.-H.. (2023). Run, don’t walk: chasing higher FLOPS for faster neural networks. Proc. IEEE/CVF Conf. Comput. Vision Pattern Recognit. 2023, 12021–12031. doi: 10.48550/arXiv.2303.03667

[B3] Dong H. Zhang Y. Gu H. Konz N. Zhang Y. Mazurowski M. A. (2023). SWSSL: Sliding window-based self-supervised learning for anomaly detection in high-resolution images. IEEE Trans. Med. Imaging. 42 (12), 3860–3870. doi: 10.1109/TMI.2023.3314318 PMC10766076 37695965

[B4] Fu Q. Yang Y. Chen X. Shang Y. (2020). Vision-based obstacle avoidance for flapping-wing aerial vehicles. Sci. China Inf. Sci. 63, 1–3. doi: 10.1007/s11432-019-2750-y

[B5] Garg D. Alam M. (2023). Smart agriculture: A literature review. J. Manage. Analytics 10, 359–415. doi: 10.1080/23270012.2023.2207184

[B6] Hu T. Wang W. Gu J. Xia Z. Zhang J. Wang B. (2023). Research on apple object detection and localization method based on improved yolox and rgb-d images. Agronomy 13, 1816. doi: 10.3390/agronomy13071816

[B7] Hui L. Lishuai Z. Yue S. Jian Z. Bian W. (2019). Real-time pedestrian detection in orchard based on improved SSD. Trans. Chin. Soc. Agric. Machinery (Transactions CSAM) 50, 29–35. doi: 10.6041/j.issn.1000-1298.2019.04.003

[B8] Ji W. Gao X. Xu B. Pan Y. Zhang Z. Zhao D. (2021). Apple target recognition method in complex environment based on improved YOLOv4. J. Food Process Eng. 44, e13866. doi: 10.1111/jfpe.13866

[B9] Ji W. Pan Y. Xu B. Wang J. (2022). A real-time apple targets detection method for picking robot based on ShufflenetV2-YOLOX. Agriculture 12, 856. doi: 10.3390/agriculture12060856

[B10] Ji W. Wang J. Xu B. Zhang T. (2023). Apple grading based on multi-dimensional view processing and deep learning. Foods 12, 2117. doi: 10.3390/foods12112117 37297365 PMC10253039

[B11] Jiansheng W. Shuguo P. Guangzhao T. Wang G. Yingchun S. (2021). Design and experiments of the binocular visual obstacle perception system for agricultural vehicles. Trans. Chin. Soc. Agric. Eng. (Transactions CSAE) 37, 55–63. doi: 10.11975/j.issn.1002-6819.2021.09.007

[B12] Li H. Li J. Wei H. Zheng L. Zhan Z. Ren Q. (2024). Slim-neck by GSConv: a lightweight-design for real-time detector architectures. J. Real-Time Image Process. 21, 62. doi: 10.48550/arXiv.2206.02424

[B13] Li S. Feng C. Liang X. Qin H. Li H. Shi L. (2018). A guided vehicle under fire conditions based on a modified ultrasonic obstacle avoidance technology. Sensors 18, 4366. doi: 10.3390/s18124366 30544725 PMC6308595

[B14] Liu Q. Lv J. Zhang C. (2024). MAE-YOLOv8-based small object detection of green crisp plum in real complex orchard environments. Comput. Electron. Agric. 226, 109458. doi: 10.1016/j.compag.2024.109458

[B15] Ming R. Jiang R. Luo H. Lai T. Guo E. Zhou Z. (2023). Comparative analysis of different uav swarm control methods on unmanned farms. Agronomy 13, 2499. doi: 10.3390/agronomy13102499

[B16] Ren S. He K. Girshick R. Jian S. (2016). Faster R-CNN: Towards real-time object detection with region proposal networks[J. IEEE Trans. Pattern Anal. Mach. Intell. 39, 1137–1149. doi: 10.1109/TPAMI.2016.2577031 27295650

[B17] Su F. Zhao Y. Shi Y. Zhao D. Wang G. Yan Y.. (2022). Tree trunk and obstacle detection in apple orchard based on improved YOLOv5s model. Agronomy 12, 2427. doi: 10.3390/agronomy12102427

[B18] Wang P. Gao M. Sun Y. Zhang H. Liao Y. Xie S. (2022). A vibration-powered self-contained node by profiling mechanism and its application in cleaner agricultural production. J. Cleaner Production 366, 132897. doi: 10.1016/j.jclepro.2022.132897

[B19] Wang J. Gao Z. Zhang Y. Zhou J. Wu J. Li P. (2021). Real-time detection and location of potted flowers based on a ZED camera and a YOLO V4-tiny deep learning algorithm. Horticulturae 8, 21. doi: 10.3390/horticulturae8010021

[B20] Yang Y. Li Y. Wen X. Zhang G. Ma Q. Cheng S.. (2022). An optimal goal point determination algorithm for automatic navigation of agricultural machinery: Improving the tracking accuracy of the Pure Pursuit algorithm. Comput. Electron. Agric. 194, 106760. doi: 10.1016/j.compag.2022.106760

[B21] Yang S. Wang W. Gao S. Deng Z. (2023). Strawberry ripeness detection based on YOLOv8 algorithm fused with LW-Swin Transformer. Comput. Electron. Agric. 215, 108360. doi: 10.1016/j.compag.2023.108360

[B22] Yang Y. Xing W. Qianglong M.. (2022). Real time planning of the obstacle avoidance path of agricultural machinery in dynamic recognition areas based on Bezier curve. Trans. Chin. Soc. Agric. Eng. (Transactions CSAE) 38, 34–43. doi: 10.11975/j.issn.1002-6819.2022.06.004

[B23] ZFrische T. Egerer S. Matezki S. Wogram J. (2018). 5-Point programme for sustainable plant protection. Environ. Sci. Europe 30, 1–17. doi: 10.1186/s12302-018-0136-2 PMC5849641 29576997

[B24] Zhang Z. Li J. Su C. Wang Z. Li Y. Li D.. (2024). A method for counting fish based on improved YOLOv8. Aquacultural Eng. 107, 102450. doi: 10.1016/j.aquaeng.2024.102450

[B25] Zhang Z. Lu Y. Zhao Y. Pan Q. Jin K. Xu G.. (2023). Ts-yolo: an all-day and lightweight tea canopy shoots detection model. Agronomy 13, 1411. doi: 10.3390/agronomy13051411

[B26] Zhang H. Zhang S. (2023). Shape-iou: More accurate metric considering bounding box shape and scale. arXiv preprint arXiv, 231217663. doi: 10.48550/arXiv.2312.17663

[B27] Zhao X. Wang K. Wu S. Wen L. Chen Z. Dong L.. (2023). An obstacle avoidance path planner for an autonomous tractor using the minimum snap algorithm. Comput. Electron. Agric. 207, 107738. doi: 10.1016/j.compag.2023.107738

